# Performance Evaluation and Improving Mechanisms of Diatomite-Modified Asphalt Mixture

**DOI:** 10.3390/ma11050686

**Published:** 2018-04-27

**Authors:** Chao Yang, Jun Xie, Xiaojun Zhou, Quantao Liu, Ling Pang

**Affiliations:** State Key Laboratory of Silicate Materials for Architectures, Wuhan University of Technology, Wuhan 430070, China; hbyangc@whut.edn.cn (C.Y.); zhouxj25@whut.edu.cn (X.Z.); liuqt@whut.edu.cn (Q.L.); lingpang@whut.edu.cn (L.P.)

**Keywords:** diatomite, styrene–butadiene–styrene (SBS) modified bitumen, diatomite-modified asphalt mixture

## Abstract

Diatomite is an inorganic natural resource in large reserve. This study consists of two phases to evaluate the effects of diatomite on asphalt mixtures. In the first phase, we characterized the diatomite in terms of mineralogical properties, chemical compositions, particle size distribution, mesoporous distribution, morphology, and IR spectra. In the second phase, road performances, referring to the permanent deformation, crack, fatigue, and moisture resistance, of asphalt mixtures with diatomite were investigated. The characterization of diatomite exhibits that it is a porous material with high SiO_2_ content and large specific surface area. It contributes to asphalt absorption and therefore leads to bonding enhancement between asphalt and aggregate. However, physical absorption instead of chemical reaction occurs according to the results of FTIR. The resistance of asphalt mixtures with diatomite to permanent deformation and moisture are superior to those of the control mixtures. But, the addition of diatomite does not help to improve the crack and fatigue resistance of asphalt mixture.

## 1. Introduction

Due to its good driving comfort, fast construction speed, convenient maintenance, and easy recycling, asphalt pavement prevails in highway engineering [1,2]. The Chinese government has been committed to developing fully the transportation industry in the past few decades. By the end of 2016, the mileage of expressways in China exceeded 130,000 km, of which more than 90% is asphalt pavement [3].

But during service periods, major damage inevitably occurs in the asphalt pavement, including rutting, cracking, and permanent deformation [4]. It is caused by the degradation of asphalt, including bonding strength breaking, high-temperature softening, low-temperature embrittlement, and heat aging [5]. 

In order to mitigate pavement damage, it is essential to improve the full temperature range performance of asphalt during the service period [6]. In recent years, a variety of modifiers, including organic and inorganic materials have been introduced. Researchers have conducted various studies to investigate their effects on the improvement of road performance. Amir [7] investigated the effect of temperature on the toughness index and fatigue properties of styrene–butadiene–styrene (SBS), a styrene-butadiene block copolymer-modified asphalt mixture created by a Universal Test Machine (UTM) apparatus. The results suggest that the SBS can increase the indirect tensile strength of an asphalt mixture at high temperatures. Taher [8] evaluated the permanent deformation characteristics of polyethylene terephthalate (PET)-modified asphalt mixtures. The results indicate that mixtures with PET modification have better resistance against permanent deformation. However, its price is too high to promote. Mahyar [4] investigated the effects of rice husk ash (RHA) as an asphalt modifier on binders and mixtures. The results suggest that the properties of the binders and mixtures were enhanced remarkably with the addition of RHA; although, the preparation process of RHA-modified asphalt is quite complex. Paravita [9] investigated the effect of crumb rubber on the properties of asphalt mixtures. The crumb rubber-modified asphalt mixture exhibited better mechanical properties. But, the modified mixture showed uncontrolled volume properties, which may affect durability. Erol [10] evaluated the effect of nano-clay materials on the enhancement of the mechanical properties of an asphalt mixture. The mixtures with nano-clay modification exhibit acceptable water damage resistance and rutting resistance except for its fatigue performance.

Nowadays, the SBS-modified bitumen is widely used. But, as for the other modifiers, they are either too expensive or show fewer improving effects according to the literature. New modifiers with low prices, easy modification procedures, and good modification effects are still in urgent demand. 

As a non-metallic mineral, diatomite is an inorganic natural resource in large reserve [11,12]. Researchers have tried to introduce it into asphalt mixtures for its rough surface, high hardness, acid and alkali resistance, wear resistance, anti-skidding, porous structure, unique component activity, stable properties, etc. [13,14]. Alejandra’s [15] research indicates that the fatigue resistance of a binder with 4% diatomite content is improved. Cong [12] investigated the physical properties, dynamic rheological behaviors, storage stability, and aging properties of different contents of modified asphalt binders. The results suggest that both viscosity and complex modulus of binders increase rapidly at high temperatures with the addition of diatomite. Compared with base asphalt binders, the resistance of modified asphalt binders to high-temperature deformation and low-temperature cracking has been greatly improved. 

Meanwhile, the pavement performances of diatomite-modified asphalt mixtures have been studied. Zhang [16] and Tan [17] evaluated the effect of diatomite on the low-temperature performance of asphalt mixtures. The results indicate that the bending strain energy density of a mixture increases with the addition of diatomite. Chen’s [18] research shows that the dynamic stability of an SBS-modified asphalt mixture is the greatest, followed by the diatomite-modified asphalt mixture and the controlled asphalt mixture. Wei [19] stated that the anti-icing performance of diatomite-modified asphalt mixtures was improved. Chen [20] suggested that the fatigue life of modified asphalt mixtures with diatomite was certainly improved under the same stress levels. Bao [21] indicates that diatomite can improve the stability and splitting strength of an asphalt mixture.

Based on the findings mentioned above, it can be found that diatomite can improve the performance of asphalt mixtures with respect to rutting resistance at high temperatures and splitting or crack resistance at low temperatures. Nevertheless, the improving mechanism necessitates further systematical research.

In this paper, X-ray Diffraction (XRD), X-ray Fluorescence (XRF), particle-size and pore-size analyzer, Scanning Electron Microscope (SEM), and Fourier-Transform Infrared Spectrometer (FTIR) tests were employed to evaluate the characteristics of diatomite. The effects of diatomite on the pavement performance of a modified asphalt mixture were also investigated. In particular, low-temperature properties were given much importance, because inorganic fillers seldom have significant effects on the low-temperature aspects. Based on the tests, we evaluated how diatomite affects the performance of asphalt mixtures.

## 2. Materials 

Base asphalt with 60–80 penetration was procured from Ezhou, China. Two SBS-modified bitumen samples were procured from Ezhou (EZ) and Inner Mongolia (IM), China, respectively. Their properties are presented in Table 1 and Table 2, respectively. Basalt aggregate and limestone filler were also included in the asphalt mixtures. The fundamental properties of diatomite and limestone powder are listed in Table 3.

## 3. Experimental Methods

### 3.1. Characteristic Methods for Diatomite and Asphalt Binder

The mineralogy, chemical composition and microscopic surface characteristic of diatomite were tested by D8 Advance X-ray Diffraction (XRD, Brooke AXS, Berlin, Germany), Axios X-ray Fluorescence (XRF, PANalytical B.V., Amsterdam, The Netherlands), and JSM-IT300 Scanning Electron Microscope (SEM, NEC Electronics Corporation, Tokyo, Japan), respectively. A Mastersizer 3000 laser particle analyzer (Malvern Instruments, Malvern, UK) was used to determine the particle size distributions of fillers.

A TriStarII3020 multi-channel ratio surface area and aperture analyzer (Micromeritics, Atlanta, GA, USA) was applied to investigate surface area and mesoporous distribution. The specific surface area was determined by the Brunauer–Emmett–Teller (BET) test method. Nitrogen was used as adsorbent, and helium or hydrogen was used as a carrier gas. The two gases were mixed at a certain proportion. When it achieved the specified relative pressure, the gas flowed through solid material. The sample was adsorbed physically by nitrogen. When the liquid nitrogen was taken away, the adsorbed nitrogen was desorbed, and a desorption peak appeared. Finally, calibration peaks were obtained by injecting pure nitrogen of known volume into the mixture. According to the peak area of the calibration peaks and desorption peaks, the adsorption amount under the relative pressure was calculated. By changing the mixing ratio of the nitrogen and carrier gas, the adsorption capacity of several nitrogen relative pressures could be determined. The specific surface area could be calculated according to the following formula:

(1) $$\frac{p}{V\left(p_{0}-p\right)}=\frac{1}{V_{m}C}+\frac{\left(C-1\right)}{V_{m}C}\frac{p}{p_{0}}$$

(2) $$S_{g}=\frac{V_{m}N_{A}A_{m}}{2240W}\times 10^{-18}$$

where *p* = partial pressure of nitrogen; *p*_0_ = saturated vapor pressure of liquid nitrogen; *V_m_* = amount of gas required to form a monolayer; *V* = total volume of adsorbed gas; *C* = constant; *S_g_* = specific surface area; *N_A_* = Avogadro constant; *A_m_* = cross-sectional area of adsorbed gas; and *W* = sample quality.

The Nicolet6700 Fourier-Transform Infrared Spectrometer (FTIR, Thermo Electron Scientific Instruments, Columbia, IN, USA) was used to obtain the IR spectra of the base asphalt, diatomite-modified asphalt binder, and diatomite. Binder specimens were made with base asphalt and 12% weight-based diatomite after constant stirring at 120 °C for 0.5 h. The test procedure was as follows: The infrared light of a certain wavelength was irradiated to the measured substance. If the radiant energy was equal to the energy level difference between the ground state and the excited state of the molecular vibration, the molecule could absorb the infrared light energy. The vibration transitioned from the ground state to the excited state. The instrument recorded the degree of infrared light absorption with the wavelength of the change function to form the infrared spectrum. When detecting the asphalt, it was dissolved in CS_2_ in a solution, and then, the KBr tablet method was used to prepare the sample. Because of the high volatility of CS_2_, the solution should be equipped with the current test. The scanning wave number range was 500~4000 cm^−1^, and the scanning frequency was 64 times.

### 3.2. Performance Evaluation of Mixtures

#### 3.2.1. Preparation of Asphalt Mixture 

**Figure 1:**
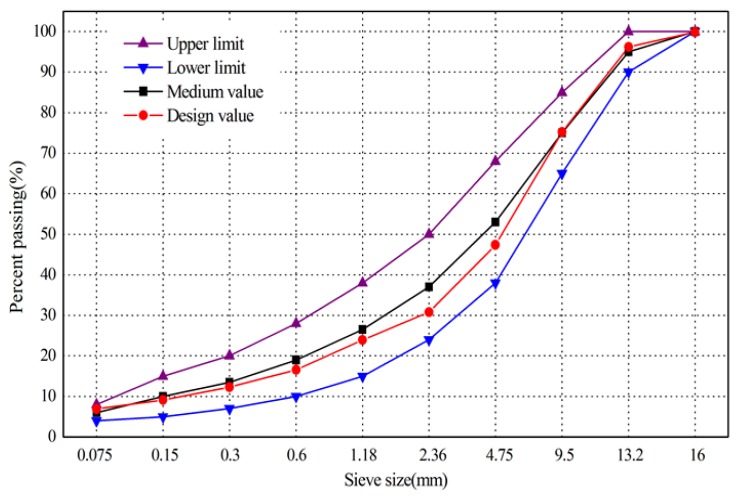
Asphalt concrete (AC)-13 gradation design used in this paper.

Four mixtures—EZ-SBS-modified, IM-SBS-modified, diatomite-modified, and base asphalt—mixtures were studied. All of them were prepared with the same gradation at optimum asphalt content. The gradation was designed with 13.2-mm nominal maximum size. Figure 1 shows the gradation. Two blending methods, namely direct and indirect blending methods, were used in the preparation of the diatomite-modified asphalt mixture. When the direct blending method was used, the diatomite was added to the mixture of asphalt and aggregate with mineral powder. When the indirect mixing method was used, the diatomite-modified asphalt binder was prepared before the preparation of the mixture [22]. Yin’s [23] research showed that the two blending methods led to approximately the same mix effect. Chen [20] determined that the optimum amount of compound diatomite modifier was 10%, while at this content, the Marshall specimens showed the best performances. Zhang [16] concluded that the optimum dosage of diatomite was 13% through the analysis of the low-temperature performance of binders and mixtures. Hence, 10–13% was a reasonable range of dosage. According to the literature review, the direct blending method was chosen, with 12% (mass ratio of diatomite and asphalt) diatomite content for the specimens’ preparation.

#### 3.2.2. Low-Temperature Performance 

**Figure 2:**
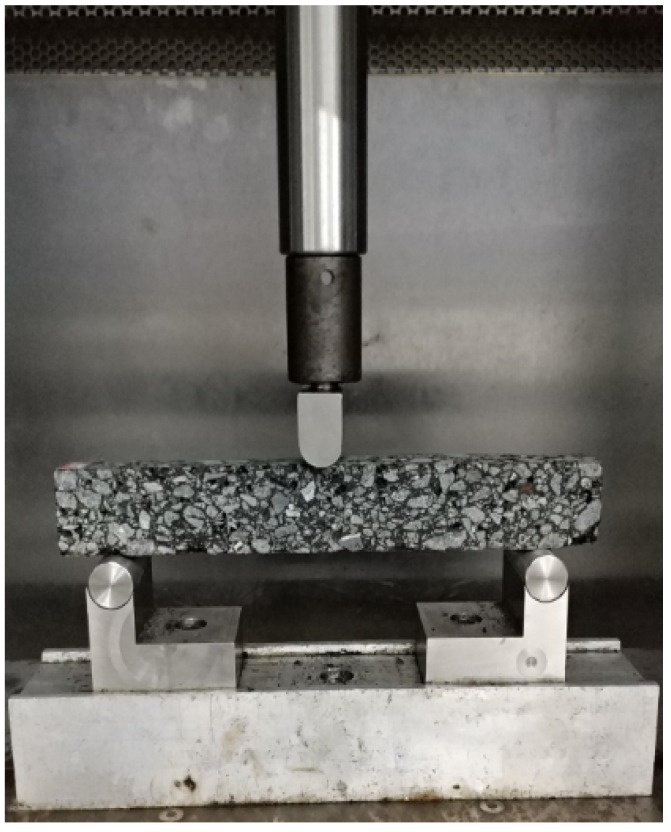
Three-points bending test set-up.

A three-points bending test is the common approach for evaluating low-temperature cracking performance of asphalt mixtures. The test device is shown in Figure 2. Beam specimens with 250 ± 2.0 mm length, 30 ± 2.0 mm width, and 35 ± 2.0 mm height were used [24]. There were five parallel specimens in each type of mixture. The test was carried out on a Universal Testing Machine (UTM)-25 from Melbourne, Australia, and the experimental parameters were −10 °C of temperature and a loading rate of 50 mm/min. The bending strain energy and bending strain energy density in this study were used to evaluate the four mixtures [25], and formulas for the calculations were as follows:

(3) $$W=\int_{s}^{s_{0}}Fds$$

where *W* = bending strain energy; *F* = force; *s* = displacement; and *s*_0_ = critical displacement.

(4) $$\frac{dw}{dv}=\int_{0}^{\varepsilon_{0}}\sigma_{ij}d\varepsilon_{ij}$$

where *dw*/*dv* = bending strain energy density; *σ_ij_* = stress component, *ε_ij_* = strain component, and *ε*_0_ = critical strain.

#### 3.2.3. High-Temperature Performance 

A rutting test is currently used to evaluate high-temperature stability. The size of the slab specimens was 300 × 300 × 50 mm. The rolling speed of the wheel was 42 times/min, and the load was 0.7 MPa. The test time was 1 h, and the test temperature was 60 °C for a standard wheel tracking test.

#### 3.2.4. Fatigue Performance

**Figure 3:**
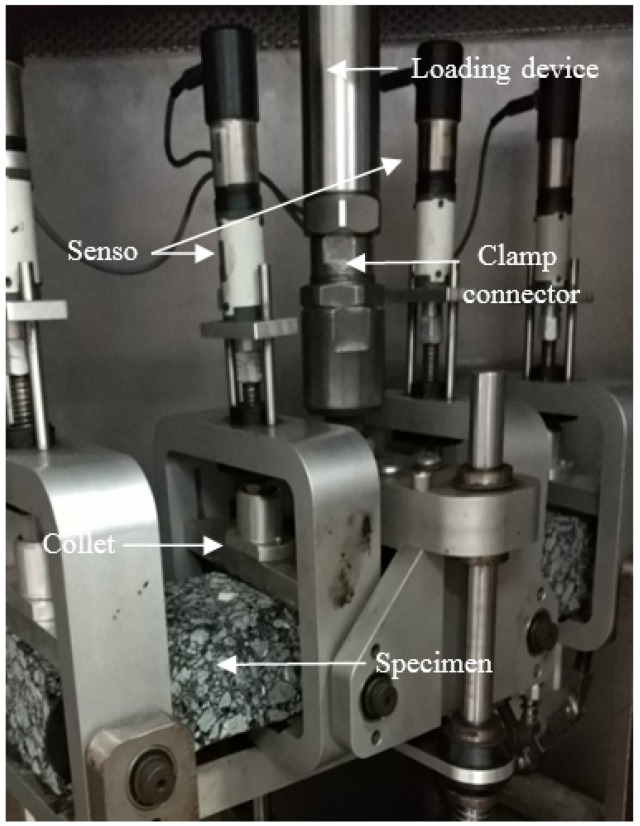
Four-points bending system for the fatigue test.

A four-points bending fatigue test was conducted by UTM-25 (IPC Global, Melbourne, Australia) as shown in Figure 3. The length, width, and height of the beam specimens were 380 ± 2.0 mm, 63.5 ± 2.0 mm, and 50 ± 2.0 mm, respectively. The test temperature was 15 °C. A haversine load pulse at 10 Hz was used. In the fatigue test, strain control loading mode was adopted to study the fatigue life of the asphalt mixture under a microstrain, such as 500 με, 600 με, 700 με, and 800 με.

#### 3.2.5. Water Stability

Water stability was used to evaluate the ability of asphalt to stripe from the aggregate surface when the asphalt mixture was subjected to water erosion. In this paper, the Marshall stability test and indirect tensile strength test were used to assess water stability.

## 4. Results and Discussion

### 4.1. Characteristics of Diatomite and Binder

#### 4.1.1. Mineralogical Properties of Diatomite and Limestone Powder

**Figure 4:**
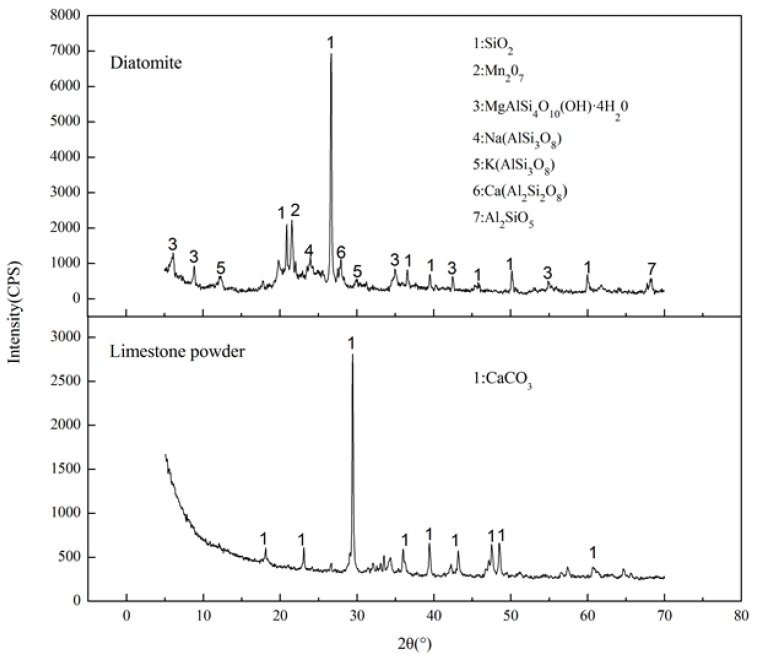
XRD pattern of the diatomite and limestone powder.

Figure 4 shows the XRD patterns of diatomite and limestone powder. A strong diffraction peak appears at 2*θ* = 26.66° in diatomite, which represents the mineral phase of SiO_2_. From the retrieved mineral composition, it can be concluded that the group OH^−^ is contained in diatomite. It is an essential reason for the surface activity and absorptivity of diatomite [26]. For the XRD pattern for limestone, there is a very strong diffraction peak of CaCO_3_ at 2*θ* = 29.43°. It implies the extremely high content of CaCO_3_.

Table 4 shows the chemical components of diatomite. Silicon (Si) displays the highest contributions, followed by Al and Fe. The content of SiO_2_ is one of the most important parameters by which to evaluate the quality of diatomite [27]. In particular, the surface of diatomite has very strong adhesion and adhesive strength due to the presence of these amorphous SiO_2_. The inert nature of SiO_2_ can also reduce the transmission speed in pavement and endow the pavement with heat insulation functions [6]. 

#### 4.1.2. Particle Size Distribution of Diatomite

**Figure 5:**
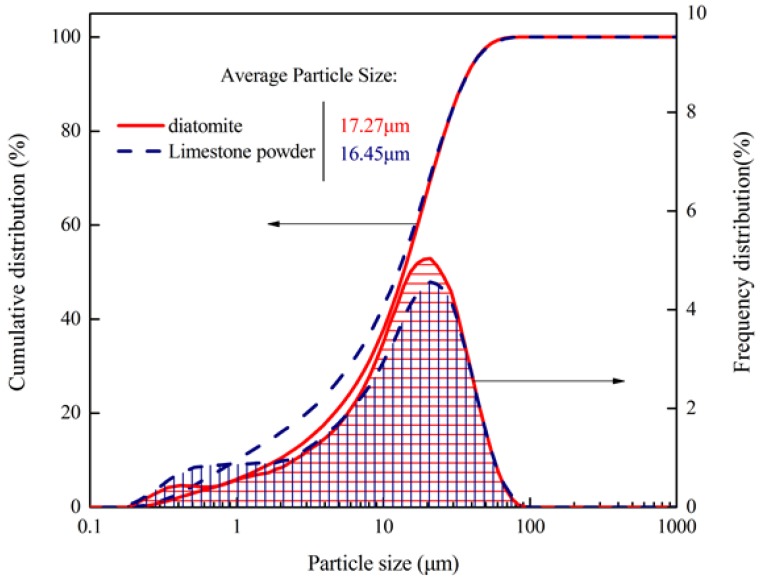
Particle size distributions of the diatomite and limestone powder.

Figure 5 shows the particle size distributions of diatomite and limestone powder. From the frequency distribution curve, it can be seen that the average particle size of diatomite is slightly bigger than that of limestone powder. In addition, their particles mainly concentrate in between 5 μm and 50 μm. Cumulative distributions results show that the proportions of particle size of diatomite less than 14.48 μm and 36.52 μm reach 50% and 90%, respectively, and for limestone powder, it is 13.27 μm and 36.63 μm, respectively. The average particle size of diatomite and limestone powder is similar. Notably the average particle size is the main factor affecting the dispersion and compatibility of filler in asphalt. It is concluded that the use of diatomite contributes to the extension and filling of asphalt for its large specific surface area. It further lays a foundation for improving the performance of the mixture.

#### 4.1.3. Mesoporous Distribution of Diatomite

**Figure 6:**
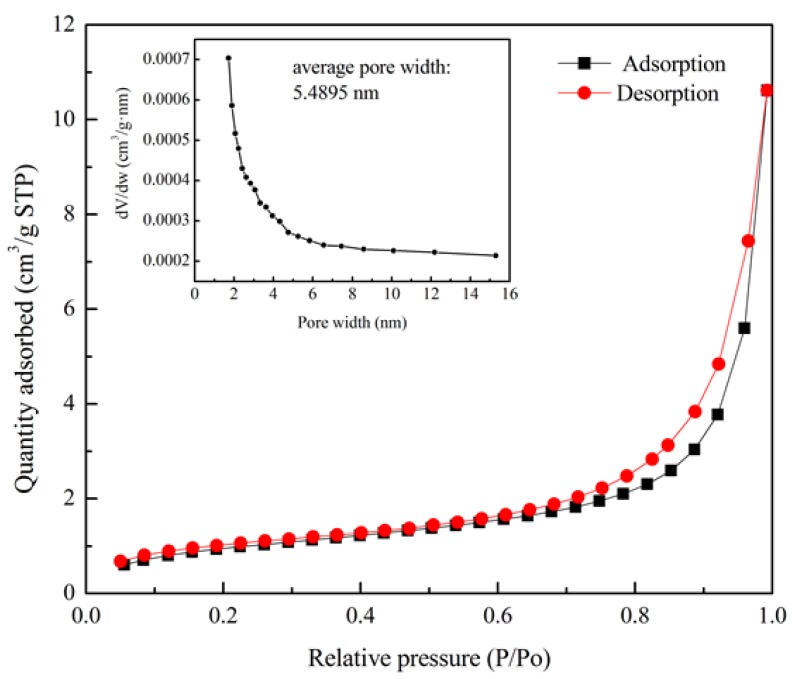
Adsorption isotherm and mesoporous distribution of diatomite.

Figure 6 illustrates N_2_ adsorption isotherms for pore size analysis. The quantity of adsorbed N_2_ increases with the increase of relative pressure, where *p* and *p*_0_ are equilibrium pressure and saturation pressure, respectively. Less adsorption in the low-pressure zone indicates that the force between the adsorbent and the adsorbate is quite weak. In the high *p*/*p*_0_ range, with the rise of pressure, the adsorption rate increases significantly. It can be observed that the desorption isotherm is above the adsorption isotherm when the relative pressure is between 0.6 and 1.0. In this interval, the adsorbate condenses in capillary, resulting in desorption hysteresis. This result confirms the conclusion made by Garderen [28], who found that diatomite is a layered structure with a narrow number of mesopores in it. The mesoporous distribution diagram is highlighted in Figure 6 by using the adsorption branch data. It can be seen that the pore size of diatomite mainly concentrates from 1 nm to 8 nm. The average pore diameter is 5.4895 nm. The mesoporous structure can subsequently enhance the capability of the absorbing light components of asphalt, resulting in the improvement of viscosity and high-temperature performance of asphalt.

#### 4.1.4. SEM Results of Diatomite

**Figure 7:**
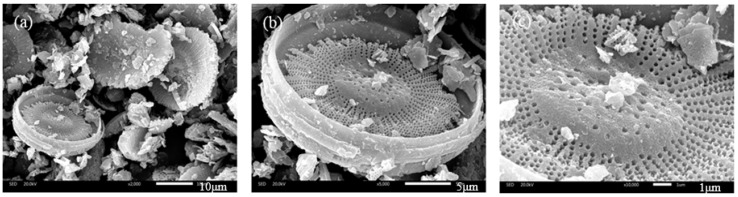
Surface microstructure of diatomite: (**a**) 2000×; (**b**) 5000×; (**c**) 10,000×.

It can be seen in the SEM images of Figure 7 that the shape of diatomite is a disc, believed to belong to cyclotella and stephanodiscus [21]. The average diameter of diatomite particles is about 20 μm. Further observation shows that there are a variety of small opening holes in the outer layer shell of diatomite. This specific structure of diatomite has certain influence on the asphalt mixture. It not only results in the large surface area of diatomite, but also facilitates the adsorption and wetting of asphalt.

#### 4.1.5. FTIR Test Results

**Figure 8:**
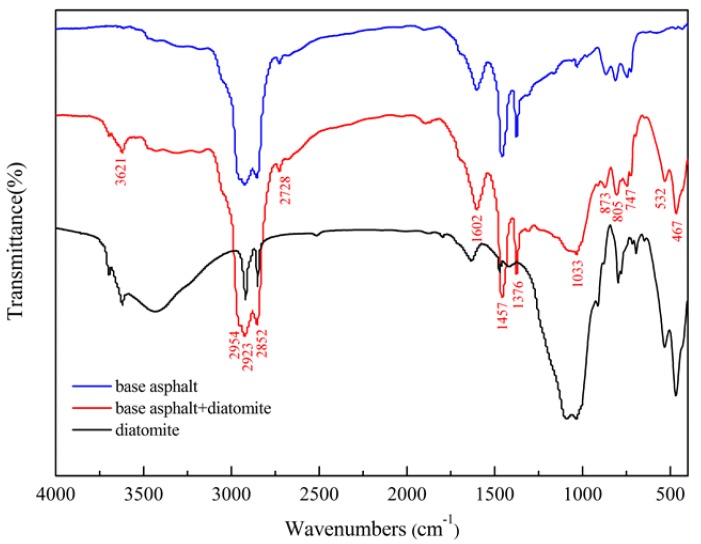
FTIR test results.

Figure 8 shows the FTIR patterns for asphalt, diatomite, and asphalt binder specimens. The results indicate that the peak at 2954 cm^−1^ is the asymmetric stretching vibrations in CH_3_. The two peaks at 2923 cm^−1^ and 2852 cm^−1^ are the asymmetric and symmetric stretching vibration in CH_2_. The peak at 2728 cm^−1^ is the C–H stretching vibration in saturated alkyl. Peak at 1602 cm^−1^ is due to the stretching vibration of the benzene ring skeleton. The peaks at 1457 cm^−1^ and 1376 cm^−1^ stand for the symmetric and asymmetric flexural vibrations in CH_3_. The absorption peaks of 873 cm^−1^ and 805 cm^−1^ are the outer flexural vibration absorption peaks of the hydrocarbon covalent bond of substituted benzene. The absorption peak at 747 cm^−1^ is the result of the alkyl flexural vibration. 

There are three new peaks for diatomic modified asphalt, compared with base asphalt. They are the vibrational peaks of water at 3621 cm^−1^, the stretching vibrations of Si–O bonds at 1033 cm^−1^, and the vibrational bands of inorganic compounds near the 500 cm^−1^, respectively. These peaks are the characteristic absorbed peaks for diatomite. It is observed that no new absorption peak appears on the spectrum of asphalt binder. Hence, it is proven that mixing of diatomite and asphalt is a simple physical blend. No new functional groups appear in the modified asphalt on account of the addition of diatomite. 

### 4.2. Performance of Asphalt Mixtures

#### 4.2.1. Results of the Three-Point Bending Test

The low-temperature performance of an asphalt mixture is determined by the tensile strength of asphalt and its combination with the aggregate. Usually, inorganic filler can improve the rutting performance of an asphalt mixture but has little effect on the improvement of the low-temperature performance, because the addition of filler tends to enhance the hardness of asphalt and increase the possibility of brittle fracture at low temperatures. Therefore, the focus falls on the influence of diatomite on the anti-cracking performance of the asphalt mixture.

**Figure 9:**
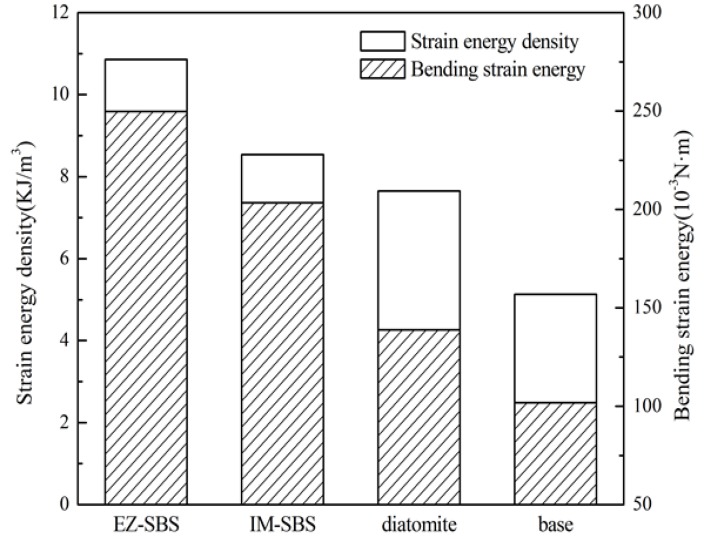
Strain energy density and bending strain energy of the four asphalt mixtures.

The test results are shown in Table 5 and Figure 9. It is seen that EZ-SBS-modified mixture shows the maximum tensile strain, strain energy density, and bending strain energy, followed by IM-SBS- and diatomite-modified mixtures. Compared with the base asphalt mixtures, the energy density and strain energy of mixtures with diatomite are improved to a certain extent. It may be ascribed to the hardening effects of asphalt adsorbed in the pores of diatomite at low temperatures, which enhanced the mechanical combination of asphalt and diatomite, further improving the low-temperature performance of diatomite asphalt mortar [29]. However, the extent of the improvement is not as great as that of SBS-modified asphalt. 

Some discussions in the literature show that low-temperature performance of diatomite-modified asphalt mixture is comparable to or slightly lower than that of polymer-modified asphalt mixtures, such as SBS [30,31]. However, the test results showed that diatomite has little effect on the improvement of the low-temperature performance of an asphalt mixture. The reason is that an inorganic substance, such as diatomite, has no cross-linking and vulcanization with asphalt, unlike the modification mechanism of asphalt by SBS. 

#### 4.2.2. Results of the Rutting Test

It can be seen from the rutting tests results in Table 6 that the EZ-SBS-modified asphalt mixture has the highest dynamic stability. The dynamic stability of the diatomite-modified asphalt mixture is larger than that of the IM-SBS-modified asphalt mixture and about 3.4 times that of the base asphalt mixture. The reason is that diatomite, with its porous structure and large surface area, can absorb the light content of asphalt, which increases the overall complex shear modulus of the asphalt mortar and improves the rutting resistance of the mixture. In addition, diatomite is an inert substance with a high content of SiO_2_, which is insensitive to the change of temperature [6]. Therefore, diatomite-modified asphalt pavement has the functions of thermal insulation and heat resistance. 

#### 4.2.3. Results of the Four-Point Bending Test

**Figure 10:**
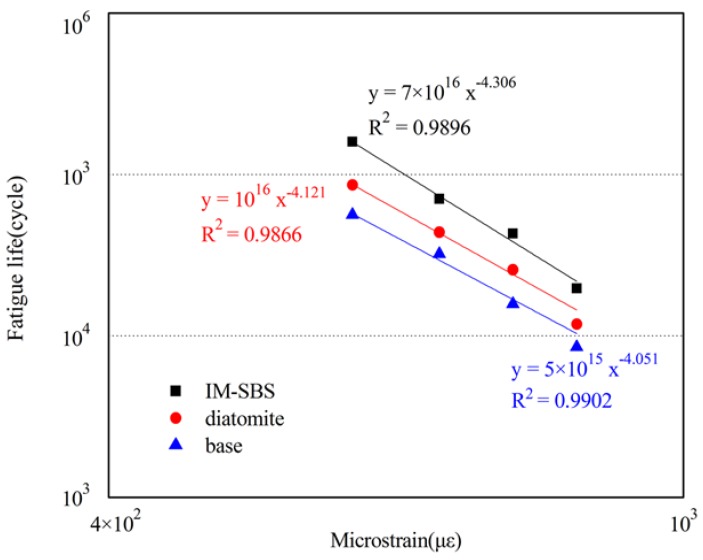
Fatigue curves of the three kinds of asphalt mixtures.

The fatigue lives of the three kinds of asphalt mixture beams specimens are shown in Figure 10. There are four microstrain levels including 500 με, 600 με, 700 με, and 800 με. It appears that the fatigue life of the IM-SBS-modified asphalt mixture is significantly greater than the other two kinds of mixtures. Meanwhile, compared with the base asphalt mixture, the fatigue performance of the asphalt mixture with diatomite is significantly improved. This is ascribed to the excellent compatibility between diatomite and asphalt, which can reduce mixing time, prevent aging of the modified asphalt, and directly improve the durability of asphalt mixture [27].

#### 4.2.4. Results of Marshall Stability and Indirect Tensile Strength Test

**Figure 11:**
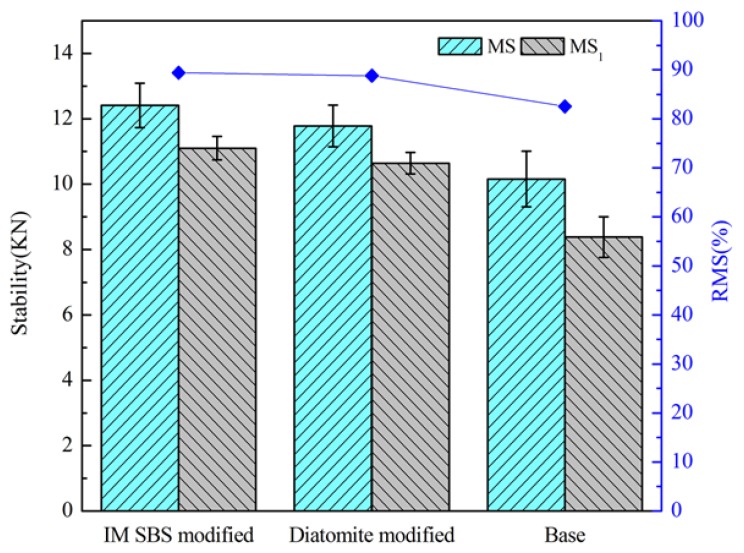
Results of the Marshall stability test.

**Figure 12:**
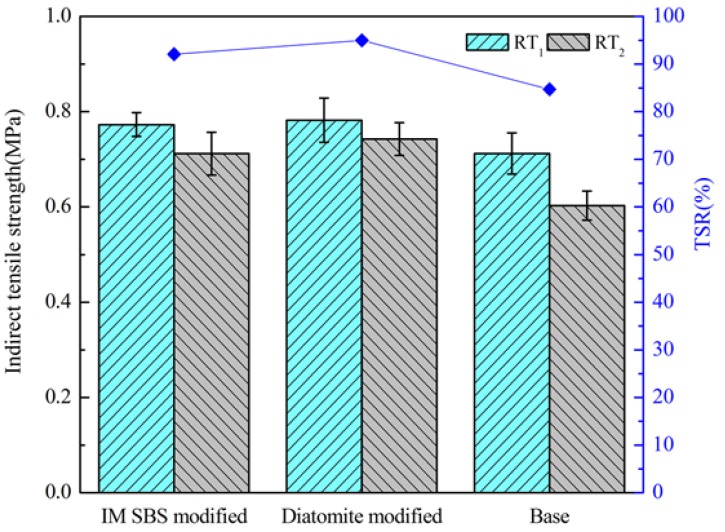
Results of the indirect tensile strength test.

The results of Marshall stability (MS), immersion Marshall stability (MS_1_), and residual Marshall stability (RMS) tests are shown in Figure 11. The results of the indirect tensile strength of normal temperature group (RT_1_), indirect tensile strength of freezing and thawing group (RT_2_), and tensile strength ratio (TSR) were shown in Figure 12. 

It can be seen that although the MS and MS_1_ of the asphalt mixture with diatomite are lower than those of the IM-SBS-modified asphalt mixture, their RMSs are approximately equal. The TSR of the diatomite-modified asphalt mixture is slightly higher than that of the IM-SBS-modified asphalt mixture. With the addition of diatomite, the parameter value of the asphalt mixture is higher than that of base asphalt mixture, leading to the increase in the cohesive force between the asphalt and the aggregate. It contributes to the increase in shear resistance and stability [32].

## 5. Conclusions

This study investigated the characteristics of diatomite by various characterization methods. The influence of diatomite as modifier on asphalt mixture was also studied by comparing the pavement performance of SBS-modified, diatomite-modified, and base asphalt mixtures. According to the above results, the following items can be concluded:

(1) The group OH^–^ was contained in diatomite. It is an essential reason for the surface activity and absorptivity of diatomite. The porous structure of diatomite improves its adhesion and wet ability with asphalt. Small particle size, numerous mesopores, and large specific surface area enhance its adsordability for the light components of asphalt. The characteristics of diatomite contribute to its strong physical connection with asphalt. They provide a possible reason for its enhancement of asphalt mixture performance.
(2) The addition of diatomite resulted in an increase in the high-temperature performance of the asphalt mixture but resulted in little improvement of the low-temperature performance. Therefore, in terms of its practical engineering application, diatomite-modified asphalt mixture is not suitable for application in the upper layer of asphalt pavement in cold areas.
(3) Although it did not perform as well as the SBS-modified asphalt mixture, the asphalt mixture with diatomite showed better fatigue performance and water stability than the base asphalt mixture. In addition, due to its low cost and simple modification process, the economic benefits of the diatomite-modified asphalt mixture have great advantages compared with the traditional modified asphalt mixture.

## Figures and Tables

**Table 1:** Measured values of base asphalt.

Indexes	Measured Values	Specification
Specific gravity	1.034	N/A
Penetration at 25 °C (0.1 mm)	63	60–80
Ductility, 5 cm/min, 15 °C (cm)	>150	≥100
Softening point (°C)	48	≥46
Apparent viscosity, 135 °C (Pa·s)	0.48	≤1.5
Loss on heating (%)	+0.09	≤±0.8

**Table 2:** Measured values of styrene–butadiene–styrene (SBS)-modified bitumen.

Indexes	Measured Values		Specification
	EZ	IM	
Specific gravity	1.032	1.039	N/A
Penetration at 25 °C (0.1 mm)	55	68	30–80
Ductility, 5 cm/min, 5 °C (cm)	56	49	≥30
Softening point (°C)	69	52	≥50
Apparent viscosity, 135 °C (Pa·s)	0.95	1.23	≤3
Loss on heating (%)	+0.32	+0.56	≤±1

**Table 3:** Fundamental properties of limestone powder and diatomite.

Indexes	Diatomite	Limestone Powder
Color	light yellow	white
Apparent density (g/cm^3^)	2.18	2.67
Water content (%)	1.81	0.55
Specific surface area (m^2^/g)	29.35	1.47
Hydrophilic coefficient	0.5	0.6

**Table 4:** Chemical components of diatomite and limestone powder.

Compound		SiO_2_	CaO	Al_2_O_3_	Fe_2_O_3_	K_2_O	MgO	TiO_2_	Loss	Others
Content (wt %)	Diatomite	62.21	0.36	12.07	4.52	1.53	1.10	0.70	15.89	1.62
	Limestone powder	1.79	55.46	0.18	0.09	-	0.52	-	41.72	0.24

**Table 5:** Low-temperature bending test results of the asphalt mixtures.

Mixtures Types	Flexural Strength (MPa)	Tensile Strain (με)	Bending Stiffness Modulus (MPa)
EZ-SBS-modified	10.127	2077.16	4996.93
IM-SBS-modified	10.481	1576.36	7229.83
Diatomite-modified	8.411	1352.72	6570.01
Base	7.910	1130.84	7333.69

**Table 6:** Rutting experiment results of asphalt mixtures.

Mixtures Types	45 min d_1_ (mm)	60 min d_2_ (mm)	Dynamic Stability (times/mm)
EZ-SBS-modified	1.229	1.295	9545
IM-SBS-modified	2.477	2.681	3088
Diatomite-modified	1.574	1.686	5625
Base	3.144	3.527	1645
